# (Methanol-κ*O*)(methano­lato-κ*O*)oxido{*N*′-[1-(2-oxidonaphthalen-1-yl-κ*O*)ethyl­idene]nicotinohydrazidato-κ^2^ *N*′,*O*}vanadium(V)

**DOI:** 10.1107/S1600536811039766

**Published:** 2011-10-05

**Authors:** Chen-Yi Wang, Juan-Juan Hu, Hai-Yu Tu, Pei-Fei Zhu, Su-Jun Sheng

**Affiliations:** a Department of Chemistry, Huzhou University, Huzhou 313000, People’s Republic of China; b Huzhou No. 11 Middle School, Huzhou 313000, People’s Republic of China

## Abstract

The title oxovanadium(V) complex, [V(C_18_H_13_N_3_O_2_)(CH_3_O)O(CH_3_OH)], was obtained by the reaction of 1-(2-hy­droxy­naphthalen-1-yl)ethanone, nicotinohydrazide and vanadyl sulfate in methanol. The V^V^ atom is six-coordinated by the *N*,*N*,*O*-tridentate Schiff base ligand, one methano­late O atom, one methanol O atom and one oxide O atom, forming a distorted octa­hedral geometry. The methanol O atom lies *trans* to the V=O group. The dihedral angle between the pyridine ring and the naphthalene ring system is 31.52 (10)°. In the crystal, inversion dimers linked by pairs of O—H⋯N hydrogen bonds occur.

## Related literature

For related Schiff base complexes, see: Wang (2009 ▶); Wang & Ye (2011 ▶). For similar oxidovanadium complexes, see: Deng *et al.* (2005 ▶); Gao *et al.* (2005 ▶); Huo *et al.* (2004 ▶).
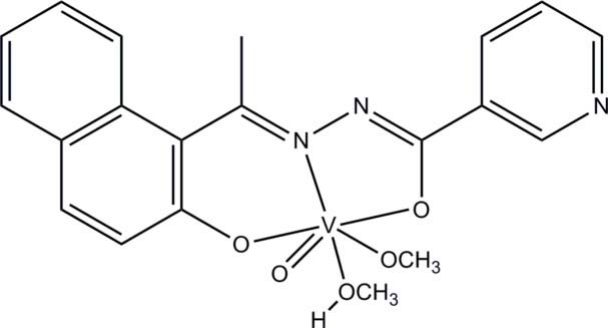


## Experimental

### 

#### Crystal data

[V(C_18_H_13_N_3_O_2_)(CH_3_O)O(CH_4_O)]
*M* *_r_* = 433.33
Triclinic, 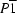

*a* = 8.056 (2) Å
*b* = 8.931 (3) Å
*c* = 14.204 (3) Å
α = 92.312 (1)°
β = 95.418 (2)°
γ = 105.481 (2)°
*V* = 978.2 (4) Å^3^
*Z* = 2
Mo *K*α radiation
μ = 0.55 mm^−1^
*T* = 298 K
0.18 × 0.17 × 0.17 mm

#### Data collection

Bruker SMART CCD diffractometer
Absorption correction: multi-scan (*SADABS*; Sheldrick, 1996 ▶) *T*_min_ = 0.908, *T*_max_ = 0.913
6564 measured reflections
4117 independent reflections
3350 reflections with *I* > 2σ(*I*)
*R*_int_ = 0.019

#### Refinement

*R*[*F* ^2^ > 2σ(*F* ^2^)] = 0.039
*wR*(*F* ^2^) = 0.101
*S* = 1.05
4117 reflections
268 parameters
1 restraint
H atoms treated by a mixture of independent and constrained refinement
Δρ_max_ = 0.24 e Å^−3^
Δρ_min_ = −0.34 e Å^−3^

### 

Data collection: *SMART* (Bruker, 1998 ▶); cell refinement: *SAINT* (Bruker, 1998 ▶); data reduction: *SAINT*; program(s) used to solve structure: *SHELXS97* (Sheldrick, 2008 ▶); program(s) used to refine structure: *SHELXL97* (Sheldrick, 2008 ▶); molecular graphics: *XP* in *SHELXTL* (Sheldrick, 2008 ▶); software used to prepare material for publication: *SHELXTL*.

## Supplementary Material

Crystal structure: contains datablock(s) global, I. DOI: 10.1107/S1600536811039766/hb6424sup1.cif

Structure factors: contains datablock(s) I. DOI: 10.1107/S1600536811039766/hb6424Isup2.hkl

Additional supplementary materials:  crystallographic information; 3D view; checkCIF report

## Figures and Tables

**Table 1:** Selected bond lengths (Å)

V1—O3	1.5826 (17)
V1—O5	1.7796 (15)
V1—O1	1.8555 (15)
V1—O2	1.9716 (15)
V1—N1	2.1143 (17)
V1—O4	2.3162 (18)

**Table 2:** Hydrogen-bond geometry (Å, °)

*D*—H⋯*A*	*D*—H	H⋯*A*	*D*⋯*A*	*D*—H⋯*A*
O4—H4⋯N3^i^	0.84 (1)	1.90 (1)	2.734 (3)	173 (3)

Symmetry code: (i) 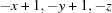.

**Fig. 1.**
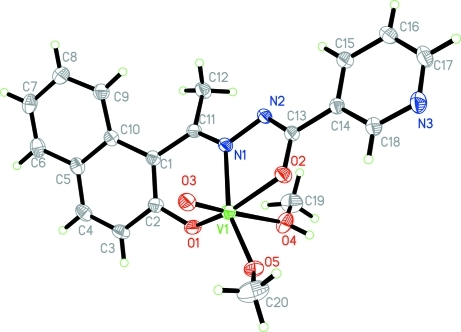
The molecular structure of the title compound, showing displacement ellipsoids drawn at the 30% probability level.

## supplementary crystallographic information

### Comment

As part of our investigations into new Schiff base complexes (Wang & Ye, 2011; Wang, 2009), we have synthesized the title compound, a new mononuclear oxovanadium(V) complex, Fig. 1. The V atom in the complex is six-coordinated by the NNO donor atoms of the Schiff base ligand, one methoxy O atom, one methanol O atom, and one oxo O atom, forming an octahedral geometry. The V–O and V–N bond lengths (Table 1) are typical and are comparable with those observed in other similar vanadium complexes (Deng *et al.*, 2005; Gao *et al.*, 2005; Huo *et al.*, 2004).

### Experimental

1-(2-Hydroxynaphthalen-1-yl)ethanone (1.0 mmol, 0.19 g), nicotinohydrazide (1.0 mmol, 0.14 g), and vanadyl sulfate (1.0 mmol, 0.16 g) were dissolved in methanol (30 ml). The mixture was stirred at room temperature for 10 min to give a clear brown solution. After keeping the solution in air for a week, brown block-shaped crystals were formed at the bottom of the vessel.

### Refinement

The methanol H atom was located from a difference Fourier map and refined isotropically, with O—H distance restrained to 0.85 (1) Å. The remaining H atoms were placed in geometrically idealized positions and constrained to ride on their parent atoms, with C—H distances in the range 0.93–0.96 Å, and with *U*_iso_(H) set at 1.2 or 1.5*U*_eq_(C).

### Crystal data

**Table:**

[V(C_18_H_13_N_3_O_2_)(CH_3_O)O(CH_4_O)]	*Z* = 2
*M**_r_* = 433.33	*F*(000) = 448
Triclinic, *P*1	*D*_x_ = 1.471 Mg m^−^^3^
Hall symbol: -P 1	Mo *K*α radiation, λ = 0.71073 Å
*a* = 8.056 (2) Å	Cell parameters from 2743 reflections
*b* = 8.931 (3) Å	θ = 2.6–28.3°
*c* = 14.204 (3) Å	µ = 0.55 mm^−^^1^
α = 92.312 (1)°	*T* = 298 K
β = 95.418 (2)°	Block, brown
γ = 105.481 (2)°	0.18 × 0.17 × 0.17 mm
*V* = 978.2 (4) Å^3^	

### Data collection

**Table:**

Bruker SMART CCD diffractometer	4117 independent reflections
Radiation source: fine-focus sealed tube	3350 reflections with *I* > 2σ(*I*)
graphite	*R*_int_ = 0.019
ω scans	θ_max_ = 27.0°, θ_min_ = 2.6°
Absorption correction: multi-scan (*SADABS*; Sheldrick, 1996)	*h* = −9→10
*T*_min_ = 0.908, *T*_max_ = 0.913	*k* = −11→11
6564 measured reflections	*l* = −18→17

### Refinement

**Table:**

Refinement on *F*^2^	Primary atom site location: structure-invariant direct methods
Least-squares matrix: full	Secondary atom site location: difference Fourier map
*R*[*F*^2^ > 2σ(*F*^2^)] = 0.039	Hydrogen site location: inferred from neighbouring sites
*wR*(*F*^2^) = 0.101	H atoms treated by a mixture of independent and constrained refinement
*S* = 1.05	*w* = 1/[σ^2^(*F*_o_^2^) + (0.0426*P*)^2^ + 0.4135*P*] where *P* = (*F*_o_^2^ + 2*F*_c_^2^)/3
4117 reflections	(Δ/σ)_max_ = 0.001
268 parameters	Δρ_max_ = 0.24 e Å^−^^3^
1 restraint	Δρ_min_ = −0.34 e Å^−^^3^

### Special details

**Table:**

Geometry. All e.s.d.'s (except the e.s.d. in the dihedral angle between two l.s. planes) are estimated using the full covariance matrix. The cell e.s.d.'s are taken into account individually in the estimation of e.s.d.'s in distances, angles and torsion angles; correlations between e.s.d.'s in cell parameters are only used when they are defined by crystal symmetry. An approximate (isotropic) treatment of cell e.s.d.'s is used for estimating e.s.d.'s involving l.s. planes.
Refinement. Refinement of *F*^2^ against ALL reflections. The weighted *R*-factor *wR* and goodness of fit *S* are based on *F*^2^, conventional *R*-factors *R* are based on *F*, with *F* set to zero for negative *F*^2^. The threshold expression of *F*^2^ > σ(*F*^2^) is used only for calculating *R*-factors(gt) *etc*. and is not relevant to the choice of reflections for refinement. *R*-factors based on *F*^2^ are statistically about twice as large as those based on *F*, and *R*- factors based on ALL data will be even larger.

### Fractional atomic coordinates and isotropic or equivalent isotropic displacement parameters (Å^2^)

**Table:**

	*x*	*y*	*z*	*U*_iso_*/*U*_eq_	
V1	0.72590 (5)	0.34810 (4)	0.26717 (2)	0.03238 (12)	
N1	0.8461 (2)	0.2127 (2)	0.18547 (11)	0.0313 (4)	
N2	0.7882 (2)	0.1918 (2)	0.08861 (11)	0.0334 (4)	
N3	0.3240 (3)	0.2650 (3)	−0.12462 (14)	0.0468 (5)	
O1	0.9408 (2)	0.40094 (18)	0.33860 (10)	0.0393 (4)	
O2	0.58351 (19)	0.29999 (18)	0.14338 (10)	0.0373 (4)	
O3	0.6236 (2)	0.20826 (19)	0.32235 (11)	0.0436 (4)	
O4	0.8772 (2)	0.5456 (2)	0.18195 (13)	0.0488 (4)	
O5	0.6277 (2)	0.49724 (18)	0.29763 (11)	0.0394 (4)	
C1	1.0365 (3)	0.1721 (3)	0.31538 (14)	0.0320 (5)	
C2	1.0262 (3)	0.3008 (3)	0.37105 (15)	0.0335 (5)	
C3	1.1149 (3)	0.3354 (3)	0.46378 (15)	0.0393 (5)	
H3	1.1147	0.4263	0.4981	0.047*	
C4	1.1993 (3)	0.2377 (3)	0.50247 (16)	0.0428 (6)	
H4A	1.2587	0.2634	0.5628	0.051*	
C5	1.1996 (3)	0.0961 (3)	0.45323 (15)	0.0375 (5)	
C6	1.2751 (3)	−0.0119 (3)	0.49719 (17)	0.0460 (6)	
H6	1.3333	0.0135	0.5579	0.055*	
C7	1.2650 (3)	−0.1525 (3)	0.45303 (18)	0.0498 (6)	
H7	1.3184	−0.2214	0.4823	0.060*	
C8	1.1733 (3)	−0.1919 (3)	0.36306 (18)	0.0495 (6)	
H8	1.1608	−0.2899	0.3338	0.059*	
C9	1.1014 (3)	−0.0885 (3)	0.31728 (16)	0.0418 (5)	
H9	1.0424	−0.1174	0.2570	0.050*	
C10	1.1148 (3)	0.0606 (3)	0.35915 (14)	0.0332 (5)	
C11	0.9751 (3)	0.1582 (2)	0.21387 (14)	0.0315 (5)	
C12	1.0689 (3)	0.0947 (3)	0.14163 (16)	0.0453 (6)	
H12A	1.0006	−0.0071	0.1172	0.068*	
H12B	1.1788	0.0882	0.1710	0.068*	
H12C	1.0870	0.1627	0.0907	0.068*	
C13	0.6477 (3)	0.2374 (2)	0.07614 (14)	0.0318 (5)	
C14	0.5526 (3)	0.2159 (2)	−0.02017 (14)	0.0315 (4)	
C15	0.5965 (3)	0.1316 (3)	−0.09313 (15)	0.0371 (5)	
H15	0.6872	0.0860	−0.0827	0.045*	
C16	0.5042 (3)	0.1159 (3)	−0.18146 (16)	0.0432 (6)	
H16	0.5321	0.0605	−0.2317	0.052*	
C17	0.3698 (3)	0.1840 (3)	−0.19387 (16)	0.0472 (6)	
H17	0.3078	0.1730	−0.2536	0.057*	
C18	0.4158 (3)	0.2800 (3)	−0.03938 (16)	0.0401 (5)	
H18	0.3858	0.3366	0.0095	0.048*	
C19	1.0283 (4)	0.5696 (4)	0.1364 (2)	0.0628 (8)	
H19A	1.1219	0.5587	0.1803	0.094*	
H19B	1.0570	0.6724	0.1141	0.094*	
H19C	1.0095	0.4941	0.0837	0.094*	
C20	0.4631 (4)	0.4778 (4)	0.3286 (3)	0.0748 (10)	
H20A	0.3755	0.4375	0.2764	0.112*	
H20B	0.4528	0.5764	0.3529	0.112*	
H20C	0.4486	0.4061	0.3778	0.112*	
H4	0.814 (4)	0.600 (3)	0.159 (2)	0.080*	

### Atomic displacement parameters (Å^2^)

**Table:**

	*U*^11^	*U*^22^	*U*^33^	*U*^12^	*U*^13^	*U*^23^
V1	0.0342 (2)	0.0342 (2)	0.0302 (2)	0.01284 (16)	0.00180 (14)	−0.00170 (14)
N1	0.0326 (9)	0.0343 (10)	0.0267 (8)	0.0111 (8)	−0.0022 (7)	−0.0020 (7)
N2	0.0369 (10)	0.0382 (10)	0.0261 (8)	0.0144 (8)	−0.0017 (7)	−0.0019 (7)
N3	0.0436 (12)	0.0538 (13)	0.0434 (11)	0.0169 (10)	−0.0063 (9)	0.0081 (9)
O1	0.0416 (9)	0.0370 (9)	0.0399 (8)	0.0169 (7)	−0.0064 (7)	−0.0082 (7)
O2	0.0356 (8)	0.0473 (10)	0.0319 (8)	0.0179 (7)	0.0009 (6)	−0.0026 (7)
O3	0.0529 (10)	0.0412 (10)	0.0363 (8)	0.0113 (8)	0.0070 (7)	0.0025 (7)
O4	0.0403 (10)	0.0485 (11)	0.0640 (11)	0.0180 (8)	0.0131 (8)	0.0204 (8)
O5	0.0347 (8)	0.0400 (9)	0.0461 (9)	0.0141 (7)	0.0082 (7)	−0.0033 (7)
C1	0.0280 (11)	0.0370 (12)	0.0307 (10)	0.0102 (9)	−0.0004 (8)	−0.0004 (9)
C2	0.0289 (11)	0.0371 (12)	0.0341 (11)	0.0097 (9)	0.0010 (8)	−0.0013 (9)
C3	0.0355 (12)	0.0458 (14)	0.0349 (11)	0.0113 (10)	−0.0010 (9)	−0.0093 (10)
C4	0.0364 (12)	0.0565 (16)	0.0313 (11)	0.0096 (11)	−0.0050 (9)	−0.0038 (10)
C5	0.0288 (11)	0.0479 (14)	0.0342 (11)	0.0083 (10)	0.0002 (9)	0.0051 (10)
C6	0.0382 (13)	0.0586 (17)	0.0407 (13)	0.0137 (12)	−0.0033 (10)	0.0101 (11)
C7	0.0482 (15)	0.0541 (16)	0.0521 (15)	0.0223 (13)	0.0008 (12)	0.0165 (12)
C8	0.0559 (16)	0.0446 (15)	0.0523 (15)	0.0216 (12)	0.0038 (12)	0.0051 (11)
C9	0.0427 (13)	0.0447 (14)	0.0393 (12)	0.0168 (11)	−0.0017 (10)	0.0000 (10)
C10	0.0262 (10)	0.0400 (12)	0.0335 (11)	0.0096 (9)	0.0024 (8)	0.0027 (9)
C11	0.0316 (11)	0.0319 (11)	0.0313 (10)	0.0110 (9)	0.0003 (8)	−0.0018 (8)
C12	0.0479 (14)	0.0608 (16)	0.0358 (12)	0.0306 (13)	0.0049 (10)	−0.0019 (11)
C13	0.0337 (11)	0.0312 (11)	0.0294 (10)	0.0080 (9)	0.0004 (8)	0.0013 (8)
C14	0.0313 (11)	0.0289 (11)	0.0318 (10)	0.0043 (9)	0.0009 (8)	0.0045 (8)
C15	0.0370 (12)	0.0378 (13)	0.0356 (11)	0.0095 (10)	0.0026 (9)	0.0006 (9)
C16	0.0467 (14)	0.0448 (14)	0.0337 (12)	0.0062 (11)	0.0014 (10)	−0.0018 (10)
C17	0.0488 (15)	0.0519 (16)	0.0341 (12)	0.0053 (12)	−0.0068 (11)	0.0067 (11)
C18	0.0432 (13)	0.0436 (14)	0.0351 (12)	0.0165 (11)	−0.0002 (10)	0.0029 (10)
C19	0.0587 (18)	0.0611 (19)	0.0673 (19)	0.0090 (14)	0.0232 (14)	0.0018 (14)
C20	0.0444 (16)	0.069 (2)	0.117 (3)	0.0212 (15)	0.0286 (17)	−0.0109 (19)

### Geometric parameters (Å, °)

**Table:**

V1—O3	1.5826 (17)	C6—H6	0.9300
V1—O5	1.7796 (15)	C7—C8	1.396 (3)
V1—O1	1.8555 (15)	C7—H7	0.9300
V1—O2	1.9716 (15)	C8—C9	1.368 (3)
V1—N1	2.1143 (17)	C8—H8	0.9300
V1—O4	2.3162 (18)	C9—C10	1.409 (3)
N1—C11	1.299 (3)	C9—H9	0.9300
N1—N2	1.399 (2)	C11—C12	1.508 (3)
N2—C13	1.301 (3)	C12—H12A	0.9600
N3—C17	1.334 (3)	C12—H12B	0.9600
N3—C18	1.340 (3)	C12—H12C	0.9600
O1—C2	1.337 (3)	C13—C14	1.482 (3)
O2—C13	1.302 (2)	C14—C18	1.382 (3)
O4—C19	1.403 (3)	C14—C15	1.384 (3)
O4—H4	0.843 (10)	C15—C16	1.379 (3)
O5—C20	1.406 (3)	C15—H15	0.9300
C1—C2	1.394 (3)	C16—C17	1.377 (3)
C1—C10	1.444 (3)	C16—H16	0.9300
C1—C11	1.469 (3)	C17—H17	0.9300
C2—C3	1.419 (3)	C18—H18	0.9300
C3—C4	1.346 (3)	C19—H19A	0.9600
C3—H3	0.9300	C19—H19B	0.9600
C4—C5	1.421 (3)	C19—H19C	0.9600
C4—H4A	0.9300	C20—H20A	0.9600
C5—C6	1.407 (3)	C20—H20B	0.9600
C5—C10	1.425 (3)	C20—H20C	0.9600
C6—C7	1.360 (4)		
			
O3—V1—O5	101.71 (8)	C9—C8—H8	119.5
O3—V1—O1	100.85 (8)	C7—C8—H8	119.5
O5—V1—O1	105.37 (7)	C8—C9—C10	121.6 (2)
O3—V1—O2	99.22 (8)	C8—C9—H9	119.2
O5—V1—O2	91.49 (7)	C10—C9—H9	119.2
O1—V1—O2	150.41 (7)	C9—C10—C5	116.9 (2)
O3—V1—N1	95.53 (8)	C9—C10—C1	123.88 (19)
O5—V1—N1	159.33 (7)	C5—C10—C1	119.1 (2)
O1—V1—N1	82.18 (7)	N1—C11—C1	119.21 (18)
O2—V1—N1	74.42 (6)	N1—C11—C12	119.49 (18)
O3—V1—O4	177.71 (7)	C1—C11—C12	121.08 (18)
O5—V1—O4	80.30 (7)	C11—C12—H12A	109.5
O1—V1—O4	79.58 (7)	C11—C12—H12B	109.5
O2—V1—O4	79.56 (7)	H12A—C12—H12B	109.5
N1—V1—O4	82.28 (7)	C11—C12—H12C	109.5
C11—N1—N2	117.13 (17)	H12A—C12—H12C	109.5
C11—N1—V1	127.25 (13)	H12B—C12—H12C	109.5
N2—N1—V1	115.38 (12)	N2—C13—O2	124.12 (18)
C13—N2—N1	107.79 (16)	N2—C13—C14	118.44 (18)
C17—N3—C18	117.3 (2)	O2—C13—C14	117.43 (18)
C2—O1—V1	125.76 (14)	C18—C14—C15	117.97 (19)
C13—O2—V1	116.38 (13)	C18—C14—C13	120.36 (19)
C19—O4—V1	134.37 (17)	C15—C14—C13	121.66 (19)
C19—O4—H4	110 (2)	C16—C15—C14	119.2 (2)
V1—O4—H4	112 (2)	C16—C15—H15	120.4
C20—O5—V1	127.10 (17)	C14—C15—H15	120.4
C2—C1—C10	118.48 (18)	C17—C16—C15	118.7 (2)
C2—C1—C11	119.09 (19)	C17—C16—H16	120.7
C10—C1—C11	122.37 (18)	C15—C16—H16	120.7
O1—C2—C1	122.17 (18)	N3—C17—C16	123.3 (2)
O1—C2—C3	117.00 (19)	N3—C17—H17	118.3
C1—C2—C3	120.7 (2)	C16—C17—H17	118.3
C4—C3—C2	120.5 (2)	N3—C18—C14	123.5 (2)
C4—C3—H3	119.8	N3—C18—H18	118.2
C2—C3—H3	119.8	C14—C18—H18	118.2
C3—C4—C5	121.5 (2)	O4—C19—H19A	109.5
C3—C4—H4A	119.2	O4—C19—H19B	109.5
C5—C4—H4A	119.2	H19A—C19—H19B	109.5
C6—C5—C4	121.2 (2)	O4—C19—H19C	109.5
C6—C5—C10	119.8 (2)	H19A—C19—H19C	109.5
C4—C5—C10	119.0 (2)	H19B—C19—H19C	109.5
C7—C6—C5	121.5 (2)	O5—C20—H20A	109.5
C7—C6—H6	119.2	O5—C20—H20B	109.5
C5—C6—H6	119.2	H20A—C20—H20B	109.5
C6—C7—C8	119.0 (2)	O5—C20—H20C	109.5
C6—C7—H7	120.5	H20A—C20—H20C	109.5
C8—C7—H7	120.5	H20B—C20—H20C	109.5
C9—C8—C7	121.0 (2)		

### Hydrogen-bond geometry (Å, °)

**Table:**

*D*—H···*A*	*D*—H	H···*A*	*D*···*A*	*D*—H···*A*
O4—H4···N3^i^	0.84 (1)	1.90 (1)	2.734 (3)	173 (3)

Symmetry codes: (i) −*x*+1, −*y*+1, −*z*.
